# Assessment of the Risk of Breast Cancer Development Applying NCI Tool among Iraqi Women

**DOI:** 10.31557/APJCP.2021.22.10.3121

**Published:** 2021-10

**Authors:** Zainab Abbas Al Talebi, Seenaa Kadhum Ali, Zinah Kadhim Kareem, Dhafer A F Al-Koofee

**Affiliations:** 1 *College of Science, University of Babylon, Iraq. *; 2 *Deptment of Chemistry, Faculty of Education for Girls, University of Kufa, Iraq.*; 3 *Manager of AL Mustensrya Family Center, Iraq. *; 4 *Deptment of Clinical Laboratory Science, Faculty of Pharmacy, University of Kufa, Iraq. *

**Keywords:** Breast cancer, bioinformatics, risk assessment tool

## Abstract

**Objective:**

As part of the bioinformatics studies, we utilized National Cancer Institute (NCI)’s Breast Cancer Risk Assessment Tool to estimate the five-year period and lifetime risk of breast cancer development among Iraqi risky women.

**Methods:**

Totally, 110 risky women aged 21-67 (mean=36±7.4) years were interviewed by a series of questions regarding the risk of breast cancer development. Moreover, 100 cases with mutation in the *BRCA1* or *BRCA2* genes were included.

**Results:**

Our results demonstrated that the patient’s estimated risk of breast cancer development during the next five years and lifetime (until the age 90 years) included 0.96% (p=0.211) and 9.97% (p=0.002), respectively being relatively low. Accordingly, the lifetime risk for the breast cancer development was significantly higher (10.38%) than that of 5-year. However, the age of patients was not significantly associated to the breast cancer development as there was no significant difference among various age groups.

**Conclusion:**

It was concluded that long-term or lifetime period plays as a significant risk factor for developing breast cancer among female patients who had had a screening episode in Iraq.

## Introduction

Cancer is one of complicated and multi-stage cell processes during which the cells lose control of normal proliferation (Ilbawi and Velazquez-Berumen, 2018; Nickson et al., 2018). The cancer is likely to be classified as the major reason of death and the most important obstacle to longevity predictable in any area worldwide in the 21^st^ century (Bray et al., 2018). Cancer is accompanied by as a set of pathways that promote the cells multiplication without natural control. Cancer can be considered as the major or second leading cause of death before age of 70 years in 91 countries, and as third or fourth most reason in 22 additional countries(Bray et al., 2018) . Breast cancer contains various subtypes in terms of morphologic structures with various risk factors, responding differently to the anticancer therapy. Around two million women have been recently recognized with breast cancer in 2018, worldwide, accounting for ¼ of total cancer cases among women. Mutations in the *BRCA1 *or *BRCA2* genes induce the progress of breast cancer. Breast cancer is the major cancer in most of countries and is considered as the main cause of deaths due to cancer in more than 100 countries (Bray et al., 2018). Breast cancer-mediated death cases has increased to be involve 2,173, or 1.23% of all deaths in Iraq, according to the WHO data published in 2017. 

Characteristic molecular subtypes of breast cancer were identified using gene expression, a process which is expensive and complex (Perou et al., 2000). Numerous risk factors such as inherent cases being family history, age, late menopause, early menarche, and sex have been stated. Other epigenetic factors are associated with an increased risk of breast cancer including alcohol consumption, menopausal obesity, the use of combined progestin and estrogen after menopause and smoking. Many risk factors affect breast tissue exposition to hormones for life. Concerning reproductive hormones, it is likely that they affect the risk of breast cancer through promotion of cancer cell growth, proliferation and increase of the likelihood of DNA damage (Kushi et al., 2012; Al-Naggar, 2013). Bioinformatics simply means applying computational techniques for understanding and organizing information related to biological macromolecules. This combination of the two subjects is largely attributed to the fact that biology itself is information technology. Simultaneously, there has been significant progress in technologies that provide raw data. According to studies by Kerlavage, the experimental lab can easily produce more than 100 GB of data per day (Magazine, 1999; Luscombe et al., 2001). Bioinformatics make it accountable for analysis, distribution, and the storage of biological information. It sometimes refers to the formation and development of algorithms, theory to solve formal, analysis of biological data, statistical techniques, and practical problems (Ouzounis, 2009; Sims, 2009; Vaidya and Dawkha, 2010; Yigitoglu et al., 2015a). The aim of this study was assessment of the risk of breast cancer development by application of NCI tool among Iraqi women patients who had had a screening episode (reference screening). 

## Materials and Methods

*Patients’ demographic data*

Herein, 110 female patients with risk of breast cancer development were included. The age of them included 21-67 (mean=36±7.4) years. They had no other complications such as diabetes, immune-compromising conditions, infections such as human immunodeficiency virus (HIV), corten-therapy and pregnancy. Moreover, all were inside the country and had same race and had no prior history of ductal or lobular carcinoma (DCIS and LCIS, respectively) or invasive carcinoma before the screening. Additionally, the familial information was not included in the Gail analyses. 

*Questionnaire of interview and provided data*

The questionnaire for risk factors assessment was filled through appointment letters for women (lifepool participants) who admitted for screening. Variables included age, race/ethnicity, a breast biopsy, age at the first live birth, and the number of first-degree relatives with breast cancer. The patients had the consent of participation in the study and the data was collected using both interview with them and their medical records and analyzed at further stage as a lifepool cohort study. The data was provided from baseline questionnaire for all participants who filled it in the time spanning of study. 

*Risk assessment using NCI’s Breast Cancer Risk Assessment Tool*

We estimated lifetime and five - year risk of developing breast cancer using NCI’s Breast Cancer Risk Assessment Tool (or Gail model). This model allows researchers to rate a woman’s risk of invasive breast cancer within the next five years and the age of 90 (lifelong risk). The tool evaluates/calculates collected data from interviews by patients including woman’s personal medical or familial history/first degree relatives (mother, sisters, and daughters) to estimate risk factors and possibility of developing breast cancer among them. This tool cannot accurately estimate the breast cancer risk for a range of conditions including patients with a previous history of invasive carcinoma, LCIS or DCIS and patients with a breast cancer-promoting mutation in BRCA1 or BRCA2. We used this tool to analyze the collected lifestyle, socio-demographic and health-related data. The Gail scores were used for evaluating their association with the future invasive breast cancer through Gail input variables (Nickson et al., 2018). 

*Statistical Methods*

As a personalized screening protocol, the NCI tool (or Gail model) calculated the risk percentage of developing breast cancer in 5 years and lifetime periods. The Gail risk scores were assigned using codes as described before to generate the risk probability during a specified year(Nickson et al., 2018). Intended for intergroup evaluation, the typically distributed variables were compared using independent t-test in the SPSS software version 20. The significant statistical level was considered as p<0.05.

## Results

The studied population included 110 female patients with risk of breast cancer development. Their age ranged 21-67 (mean=36±7.4) years (Table1). 

Depending on the obtained results, the mean patients’ evaluated risk of invasive breast cancer over the next five years included 0.96% which was not significantly correlated with the breast cancer development (0.211, Table 2).

Furthermore, there was a significant higher relation (p<0.005) between the mean patients’ risk of lifetime (to age of 90) and the breast cancer development in the carcinoma group compared to that of five year risk, though being relatively low (Table 3).

Table 5 revealed multiple comparisons (LSD) between the womens’ age at the time of first menstrual period where the results represented significant relation between-group in average risk while no significance finding between-group in five-year risk.

In addition, as revealed in the Table 6, there was no significant difference between the womens’ age at the time of first menstrual period regarding the average and lifetime risk using multiple comparisons (LSD). 

**Table1:** The Demographic Data of Participants

Characters	No. (%)
Age range (years)	
21-30	6 (0.05)
31-40	19 (17)
41-50	60 (54)
>50	14 (12)
Occupation	61 (55)
Unemployed	17 (15)
Private employee	27 (24)
Government officer	5 (0.04)
Education	
No education	8 (0.07)
Primary school	4 (0.01)
High school/Diploma	12 (11)
Bachelors’ degree	70 (6)
Master or doctor degree	16 (14)
Family income	
High	5 (0.04)
Moderate	95 (86)
low	10 (0.09)
Race/ethnicity	Arab
breast biopsy	23 (21)
Age at the first live birth	
<20	22 (20)
20-24	44 (4)
25-29	30 (27)
>=29	7 (0.06)

**Table 2:** Five-Year Risk Factors (%) Sssociated with Breast Cancer Development

Group	N	Mean	STD	Std. Error	95% CI		p value
					Lower	Upper	0.211
Risk value	92	0.96	0.67	0.07	-0.26849	0.05979	
Average risk		1.06	0.42	0.04	-0.26868	0.05999	

STD, standard deviation; N, number; CI, confidence interval

**Table 3:** The Lifetime Risk (%) of Developing Breast Cancer among Predisposed/Risky Patients

Group	N	Mean	SD	Std. Error	95% CI		p-value
					Lower	Upper	
Patients’ risk	92	9.97	4.52	0.47	-2.35521	-0.42087	0.005
Average risk		11.35	1.27	0.13	-2.35995	-0.41613	

N, number; SD, standard deviation; CI, confidence interval

**Table 4:** Correlation Analyses between Age and Breast Cancer Development

Patients	5 – year risk				Lifetime risk			
Age	Average risk		Patients’ risk		Average risk		Patients’ risk	
	P-value	r-value	P-value	r-value	P value	r-value	P-value	r-value
Significance	0	0.422**	0.037	0.218*	0.019	-0.244*	0.376	-0.093

**, Correlation is significant at the 0.01 level (2-tailed); *, Correlation is significant at the 0.05 level (2-tailed).

**Table 5:** Multiple Comparisons (LSD) between the Age Groups Regarding 5-Year Risk (%) of Developing Breast Cancer

Dependent Variable	(I) age	(J) age	Mean Difference (I-J)	Std. Error	Sig.	95% Confidence Interval	
						Lower Bound	Upper Bound
Average risk	7-11	12-13	-0.45957*	0.1737	0.01	-0.8061	-0.1130
		14-90	-0.52222*	0.19098	0.008	-0.9032	-0.1412
	12-13	7-11	0.45957*	0.1737	0.01	0.113	0.8061
		14-90	-0.06265	0.11884	0.6	-0.2997	0.1744
	14-90	7-11	0.52222*	0.19098	0.008	0.1412	0.9032
		12-13	0.06265	0.11884	0.6	-0.1744	0.2997
5-year risk	7-11	12-13	-0.16140	0.29231	0.583	-0.7445	0.4217
		14-90	-0.31825	0.32138	0.326	-0.9594	0.3229
	12-13	7-11	0.1614	0.29231	0.583	-0.4217	0.7445
		14-90	-0.15686	0.19999	0.436	-0.5558	0.2421
	14-90	7-11	0.31825	0.32138	0.326	-0.3229	0.9594
		12-13	0.15686	0.19999	0.436	-0.2421	0.5558

*, The mean difference is significant at the 0.05 level.

**Table 6:** Multiple Comparisons (LSD) between the Age Group in Lifetime Risk (%) of Developing Breast Cancer

Dependent Variable	(I) age	(J) age	Mean Difference (I-J)	Std. Error	P value	95% Confidence Interval	
						Lower Bound	Upper Bound
Average risk	7-11	12-13	0.72796	0.54861	0.189	-0.3665-	1.8224
		14-90	0.62063	0.60319	0.307	-0.5827	1.824
	12-13	7-11	-0.72796	0.54861	0.189	-1.8224	0.3665
		14-90	-0.10733	0.37535	0.776	-0.8561	0.6415
	14-90	7-11	-0.62063	0.60319	0.307	-1.8240	0.5827
		12-13	0.10733	0.37535	0.776	-0.6415	0.8561
Lifetime risk	7-11	12-13	3.37781	1.91879	0.083	-0.4501	7.2057
		14-90	3.27143	2.10966	0.126	-0.9372	7.4801
	12-13	7-11	-3.37781	1.91879	0.083	-7.2057	0.4501
		14-90	-0.10638	1.3128	0.936	-2.7254	2.5126
	14-90	7-11	-3.27143	2.10966	0.126	-7.4801	0.9372
		12-13	0.10638	1.3128	0.936	-2.5126	2.7254

*, The mean difference is significant at the 0.05 level.

## Discussion

It is predestined that 5- 10 % of breast cancer cases are caused by inherited mutations such as those in the *BRCA1* and *BRCA2* breast cancer allergy genes. This type of mutation is found in approximately less than 1% of the general population but mostly occurs in specific ethnic groups such as Eastern Europe (Schwartz et al., 2009). Results of studies about breast cancer risk in women with these mutations by age 70 have been in range of 44 -78% of women with BRCA1 mutations and 31 -56 % with BRCA2 mutations with increased breast cancer (Antoniou et al., 2003; Chen and Parmigiani, 2007). Approximately 15-20% of familial breast cancer cases are attributed to the *BRCA1* or *BRCA2* gene mutations (Turnbull and Rahman, 2008). 

DCIS is a group of unusual breast changes begin in the cells lining the breast canals. DCIS is a non-enlarged form of breast cancer because of lack of increase in nontypical cells size in the cell layer in which they develop. It was considered to be the most prevalent type of breast cancer on-site accounting nearby 83% of cases diagnosed during 2006-2010. Indeed, several of these cancers grow very tardily not influencing women’s health even if left untreated. Studies have outlined that ≥one- third of DCIS cases will develop into invasive cancer if neglected or left untreated (Allred, 2010). LCIS is cancer-like tumor and an indicator of developing the risk of invasive cancer. During 2006-2010 LCIS prevalence was diagnosed and found to be significantly less prevalent than DCIS, where it accounted for approximately 12% of breast cancers on site (Al-Naggar, 2013). Alhough rarely developing into invasive cancer, patients with LCIS are predisposed 7 -12 times higher to progress invasive cancer in each breast than those with the absence of LCIS (Kilbride and Newman, 2010). LCIS usually does not appear using a mammogram and is usually detected through a biopsy taken for other causes. Since both of these stages are pre-cancerous lesions of the breast and require cancer-oriented treatment, it is necessary to distinguish between pure LCIS from DCIS and pleomorphic LCIS (Al-Naggar, 2013). 

In previous studies, the NCI Breast Cancer Risk Assessment validity has been confirmed in multiple settings, including, among women at high risk, representing accurate results regarding the number of breast cancer cases observed at different time intervals (Bondy et al., 1994; Spiegelman et al., 1994; Costantino et al., 1999; Rockhill et al., 2001; Bondy and Newman, 2006; Decarli et al., 2006; Graubard et al., 2010). This site does not integrate with second-degree relatives, including the age of onset of breast cancer in relatives or paternal kin, so the ACS guidelines did not advise it to evaluate the individual patient’s life during the annual MRI examination, BRCAPRO or other models that are greatly influenced by family history (Parmigiani et al., 2007). In the Claus et al., (1993) Boadicea (Antoniou et al., 2008; Elsayegh et al., 2016) and the Tyrer-Cuzick (2012) models, the calibration was not tested in the general population. In another study, Amir et al., (2003) provided overall review of these models. A small study in a group of 64 high-risk clinics only found that the expected number of cases corresponded to the cases observed for the Tyrer-Cuzick model, but these expectations were somewhat low for the NCI breast cancer risk tool being Brcapro and Claus model. A group of studies that compared several scenarios, including families with affected relatives, outlined that the Tyrer-Cuzick model and breast cancer risk assessment tool commonly accord the highest risks in families with at least one affected relative, and Bracapro gave the lowest risk (Gail and Mai, 2010; Euhus et al., 2002) and another research comparing Claus Model and the NCI breast cancer risk assessment tool in 491 women with a family history of breast cancer, found that the average lifetime risk was higher in the breast cancer risk assessment tool (13.2%) compared to the Claus Model (11.2%). Previous studies have illustrated that Brcapro and the Claus Model usually has lower risks than the NCI breast cancer risk assessment tool in a high-risk clinic population (McTiernan et al., 2001). In a previous study, atypical hyperplasia has not been reported in the NHIS (National Health Survey Interview), which can cause to decrease the risk of breast cancer. For these reasons, the study has shown that the breast cancer risk assessment tool is beneficial for estimating the convergent number of women patients with a lifetime risk of ≥ 20% in the general people, though their calculations can reduce the real number (Sørlie et al., 2001; Sotiriou et al., 2003; Lønning et al., 2007). 

In our study, according to NCI’s Breast Cancer Risk Assessment Tool calculations, there was no significant association between patients and the average risk of developing breast cancer for five years. This model offered that the average risk for women was lower than that of same age and race/ethnicity for patients in the general U.S. population in 5 years. However, the average lifetime risk of developing breast cancer was significantly associated with patients’ conditions.

Bioinformatics is critical in the field of genetic pharmacology and necessary to improve precise tools for active treatment based on the tumor biology of all patients (Paik et al., 2004; Simon, 2005). Using appropriate bioinformatics tools, these findings predict risk factors, demonstrate drug resistance, provide deeper knowledge and understanding of treatments and predict risk factors, open new fields and goals and vital and disease-related signs to those beneficial drugs. Noticeably, the impact of epigenetic factors on the etiology of breast cancer is inevitable. Although a set of research related to DNA methylation, those revealed various patterns including oncogenes and tumor suppressor genes, a small portion of them link genome data with transcriptome (Minning et al., 2014). Huge developments that have occurred both in bioinformatics and their application are largely probable by multidisciplinary teams seeking depth and focused study. The combination of tools, methodologies, specificity, databases, and sensitivity must be assessed in a perfect matter. Furthermore, the results confirmed many molecular techniques before translating into clinical practice (Yigitoglu et al., 2015b). In our study we observed that the age of women patients was significantly correlated to the average, patients’ 5 – year and Lifetime risk as shown in Table 3. The results of these comparasions illustrated significant value between group in average risk. However, among patients with mutation in the* BRCA1 *and *BRCA2* genes, there was no significant association regarding neither 5-year nor the lifetime risk with the breast cancer development. 

In conclusion, the NCI site was suitable for cases of breast cancer except those related to (LCIS, DCIS, BRCA1, and BRCA2). The results illustrated that the patients risk was relatively low. Lifetime but not 5 years risk was significantly associated with developing breast cancer. 

## Author Contribution Statement

The authors confirm contribution to the paper as follows: study conception and design: Dhafer A. F. Al-Koofee; data collection: Zainab Abbas Al Talebi; analysis and interpretation of results: Seenaa kadhum Ali, Zinah Kadhim Kareem; draft manuscript preparation: Dhafer A.F. Al-Koofee; All authors reviewed the results and approved the final version of the manuscript.
